# Long-term adjuvant imatinib treatment for a patient who underwent complete resection of a localized recurrent gastrointestinal stromal tumor after preoperative imatinib treatment: A case report

**DOI:** 10.1097/MD.0000000000014477

**Published:** 2019-02-08

**Authors:** Welda E.H. Tjhoi, Kai Li, Chun-hui Shou, Wei-li Yang, Ji-ren Yu

**Affiliations:** Department of Gastrointestinal Surgery, The First Affiliated Hospital, Zhejiang University School of Medicine, Hangzhou, China.

**Keywords:** gastrointestinal stromal tumor, imatinib, long-term treatment, recurrent, surgery

## Abstract

**Rationale:**

The efficiency and tolerance of long-term adjuvant imatinib treatment for patient who underwent complete resection of a localized recurrent gastrointestinal stromal tumor (GIST) was unknown.

**Patient concerns:**

A 45-year-old man underwent complete resection of an intestinal GIST in August 2001. Four years later, a giant (11 × 8 × 6 cm) recurrent GIST located in the retroperitoneum was detected.

**Diagnosis:**

The recurrent tumor was positive for CD117 by immunohistochemistry.

**Interventions:**

The recurrent tumor was completely resected after 4 months of effective imatinib treatment (400 mg/day), and the patient continued imatinib treatment postoperatively. In June 2011, imatinib treatment was stopped for 3 weeks because of hepatitis B infection, and resumed with a reduced dose level of 300 mg/day when liver function recovered. In March 2017, imatinib treatment was interrupted again for 12 days because the patient underwent cholecystectomy.

**Outcomes:**

In December 2017, a computed tomography scan showed no signs of tumor recurrence. To date, the patient has been under adjuvant imatinib treatment for >12 years without severe side effects. The plasma concentration of imatinib (detected in February 2018) was trough concentration (C_min_) 1015.7 ng/mL and peak concentration (C_max_) 1550.5 ng/mL.

**Lessons:**

This case report highlights the active role of long-term (>12 years) imatinib treatment after complete resection of localized recurrent GIST.

## 1 Introduction

Surgical resection without residual tumor remains the main treatment for gastrointestinal stromal tumor (GIST),^[1]^ but patients with high-risk features still suffer high recurrence rates post-operatively before imatinib treatment.^[2]^ Therefore, patients with high-risk GIST need adjuvant imatinib treatment after surgery.^[1]^ For metastatic/recurrent GIST, imatinib significantly improves survival time and is considered first-line therapy until tumor progression.^[3,4]^ A recent study investigated the survival benefit of imatinib combined with surgery for localized metastatic/recurrent GIST.^[5]^ However, the results were inconsistent. Here, we report a 45-year-old male patient diagnosed with a recurrent GIST who received complete resection of the tumor after effective imatinib treatment, and has been continuously under adjuvant imatinib treatment for >12 years.

## 2 Case presentation

A 45-year-old man was diagnosed with small intestinal GIST and underwent surgery in August 2001. The tumor was found on the proximal jejunum with a size of 13 × 9 × 7 cm. The tumor mainly consisted of spindle cells with a mitotic count of 8/50 high-power fields (HPFs) by histopathology, and was positive for CD117 by immunohistochemistry. The patient did not receive adjuvant imatinib treatment after surgery. In March 2005, a giant tumor that invaded the hilus of the left kidney and left adrenal was found by a computed tomography (CT) scan during follow-up. The tumor size was about 11 × 8 × 6 cm, and biopsy showed features similar to those of the previous tumor. Thus, the diagnosis of tumor recurrence was established. After 4 months of preoperative imatinib treatment (400 mg/day), the recurrent tumor was completely resected.^[6]^ Then the patient received adjuvant imatinib treatment with a dose level of 400 mg/day. The successful treatment of this case was reported in 2007.^[6]^ Follow-up was performed every 3 to 6 months including complete blood count, chemistry profile, tumor markers, CT scan, and ultrasonic examination.

In June 2011, the patient was admitted to our hospital because of yellow discoloration of urine, fatigue, and poor appetite. A urine test showed positive urobilinogen (140 μM/L), urine protein (0.5 g/L), and urobilirubin (8.5 μM/L). A liver function test revealed increased levels of alanine aminotransferase (1103 U/L), aspartate aminotransferase (394 U/L), total bilirubin (37.0 μM/L), indirect bilirubin (21 μM/L), direct bilirubin (16 μM/L), and gamma-glutamyl transferase (322 U/L). The hepatitis B markers HBsAg, HBcAb, and HBeAg were also remarkably increased to 545.01 ng/mL, 126.26 PEIU/mL, and 138.514 PEIU/mL, respectively. A diagnosis of acute viral hepatitis B infection was established. Therefore, liver protection and the antiviral drug Entecavir (0.5 g/day) were prioritized and adjuvant imatinib treatment was interrupted for 3 weeks until liver function completely recovered. Adjuvant imatinib treatment was resumed with a reduced dose level of 300 mg/day in consideration of immunosuppression.

Because of worsened post-satiety abdominal distension, the patient received an abdominal CT scan and ultrasonography in February 2017. The CT scan showed chronic cholecystitis, gallbladder stones, and common bile duct stones. In March 2017, the patient was admitted to our hospital. Endoscopic retrograde cholangiopancreatography was performed to remove muddy stones of the common bile duct. After 1 week, laparoscopic cholecystectomy was performed. Imatinib treatment was interrupted again during the 12 days of the hospital stay. Since discharge from the hospital, the patient has been taking imatinib (300 mg/day).

The functional assessment of cancer therapy-general (FACT-G) version 4 questionnaire,^[7]^ which encompasses 4 primary dimensions of quality of life (physical well-being [PWB], social/family well-being [SWB], emotional well-being [EWB], and functional well-being [FWB]), was conducted to assess the quality of life of the patient. The total FACT-G score is obtained by summing individual subscale scores (PWB + SWB + EWB + FWB). The total FACT-G score before imatinib treatment was 50 points (PWB 18 points + SWB 24 points + EWB 3 points + FWB 5 points) and increased to 57 points (PWB 1 point + SWB 24 points + EWB 4 points + FWB 28 points) after imatinib treatment. Furthermore, the plasma concentration of imatinib was tested when available. The results on February 13, 2018 showed trough concentration (C_min_) of 1015.7 ng/mL and peak concentration (C_max_) of 1550.5 ng/mL. According to the tolerance assessment, the patient did not suffer any major side effects from long-term (>12 years) imatinib treatment except for visibly pale skin.

The patient has permitted and provided informed consent for the publication of his medical data.

## 3 Discussion

Imatinib treatment is clinically effective for GIST patients who develop tumor metastasis or recurrence. The median overall survival (OS) for metastatic/recurrent GIST was improved from 19 months to about 5 years after imatinib treatment.^[8]^ Although most patients with metastatic/recurrent GIST benefited from imatinib treatment, the median time to imatinib resistance was about 24 months.^[9]^ Resistance may be caused by pharmacokinetic changes or secondary mutations because of prolonged exposure to imatinib.^[10]^ In recent years, some researchers have investigated the role of surgery in patients with localized metastatic/recurrent GIST. One retrospective study showed that patients who underwent surgery after effective imatinib treatment had longer OS compared to those who were treated with imatinib alone (median OS: 87.6 months vs 59.9 months).^[11]^ Park et al^[12]^ performed propensity score analyses to compare the clinical outcomes of combining surgery with imatinib treatment to imatinib treatment alone in patients with metastatic/recurrent GIST who were responsive to imatinib, and they showed that surgical resection of residual lesions is likely to be effective. As shown in other studies, the prognostic factors for patients with metastatic/recurrent GIST who underwent surgery were the preoperative response to imatinib, resection status (R2 vs R0/R1), and the number of metastatic sites.^[13]^ In this case report, the patient benefited from complete resection of localized GIST followed by long-term (>12 years) adjuvant imatinib treatment, which is consistent with the studies described above. One possible explanation is that surgery for residual disease could reduce the probability of resistant clones.^[10]^

In this case, the recurrent tumor size was large (11 × 8 × 6 cm) and the location (involving the left kidney and left adrenal gland) was not suitable for resection. After 4 months of effective imatinib treatment, the recurrent tumor was successfully shrunk by 50% and was completely resected. Subsequently, the patient received long-duration adjuvant imatinib treatment. It has been recommended that high-risk GIST patients receive adjuvant imatinib treatment for at least 3 years after surgery based on the results of the SSG XVIII/AIO study.^[14]^ A study by Lin et al^[15]^ showed an improved survival rate through prolongation of adjuvant imatinib treatment for >5 years in high-risk GIST patients. Recently, the PERSIST-5 study also demonstrated that high-risk GIST patients could benefit from 5 years of adjuvant imatinib treatment (5-year RFS was 90%, and 5-year OS was 95%).^[16]^ However, the duration of imatinib treatment for localized recurrent GIST after surgery has not been determined. This patient has been under adjuvant imatinib treatment for >12 years, and long-term follow-up showed no signs of tumor recurrence. Whether the imatinib treatment can be discontinued is under discussion.

It has been reported that hepatitis B virus can be reactive during imatinib treatment, which might correlate with the immunosuppressive activities of the drug.^[17,18]^ The possible mechanisms were as follows: imatinib may induce neutropenia,^[19]^ imatinib may inhibit regulators of T-cell activation,^[20]^ and imatinib may reduce the capability of dendritic cells.^[21]^ One notable situation in this case report is exposure to hepatitis B virus infection during imatinib treatment, with the dose level of imatinib reduced to 300 mg/day. Therefore, for GIST patients who are hepatitis B carriers, antiviral treatment should be given along with the continuation of imatinib treatment.

Several studies have evaluated the correlation of the imatinib plasma concentration with clinical benefit in GIST patients. Demetri et al^[22]^ suggested that C_min_ <1100 ng/mL was associated with a shorter time to progression in patients treated with imatinib 400 or 600 mg/day. Another study found that C_min_ >760 ng/mL was associated with a 65% reduction in the risk for progression in unresectable/metastatic GIST patients treated with imatinib 400 mg/day.^[23]^ A higher imatinib plasma concentration is associated with increased side effects. For those who cannot tolerate the standard imatinib dose (400 mg/day), dose reduction (300 mg/day) can be one of the most common solutions. Yin et al investigated efficiency and safety in patients treated with low-dose imatinib (300 mg/day). They found that imatinib plasma C_min_ at 300 mg/day was 1559 ± 478 ng/mL, which was comparable to that in patients who were treated with 400 mg/day (C_min_ 2262 ± 1009 ng/mL).^[24]^ In this case, long-term follow-up showed no signs of tumor recurrence. Although the C_min_ value of 1015.7 ng/mL was slightly below the threshold range given above, it might indicate that the imatinib plasma concentration was effective.

In conclusion, this case report highlights the active role of long-term (>12 years) imatinib treatment after complete resection of localized recurrent GIST.

## Author contributions

**Data curation:** Kai Li.

**Investigation:** Ji-ren Yu.

**Methodology:** Ji-ren Yu.

**Validation:** Ji-ren Yu.

**Writing – original draft:** Welda E.H. Tjhoi.

**Writing – review & editing:** Kai Li, Chun-hui Shou, Wei-li Yang, Ji-ren Yu.
